# LncRNA H19 promotes the proliferation of pulmonary artery smooth muscle cells through AT_1_R via sponging let-7b in monocrotaline-induced pulmonary arterial hypertension

**DOI:** 10.1186/s12931-018-0956-z

**Published:** 2018-12-14

**Authors:** Hua Su, Xiaoling Xu, Chao Yan, Yangfeng Shi, Yanjie Hu, Liangliang Dong, Songmin Ying, Kejing Ying, Ruifeng Zhang

**Affiliations:** 1 Department of Respiratory Medicine, Sir Run Run Shaw Hospital, Zhejiang University School of Medicine, No. 3 Qingchun Road East, Zhejiang, Hangzhou China; 2 Department of Respiratory and Critical Care Medicine, Second Affiliated Hospital, Zhejiang University School of Medicine, No. 88 Jiefang Road, Zhejiang, Hangzhou China

**Keywords:** LncRNA, H19, miRNA, Let-7b, Pulmonary artery hypertension, AT_1_R

## Abstract

**Background:**

Pulmonary arterial hypertension (PAH) is related to inflammation, and the lncRNA H19 is associated with inflammation. However, whether PDGF-BB-H19-let-7b-AT_1_R axis contributes to the pathogenesis of PAH has not been thoroughly elucidated to date. This study investigated the role of H19 in PAH and its related mechanism.

**Methods:**

In the present study, SD rats, C57/BL6 mice and H19−/− mice were injected with monocrotaline (MCT) to establish a PAH model. H19 was detected in the cytokine-stimulated pulmonary arterial smooth muscle cells (PASMCs), serum and lungs of rats/mice. H19 overexpression and knockdown experiments were also conducted. A dual luciferase reporter assay was used to explore whether let-7b is a sponge miRNA of H19, and AT_1_R is a novel target of let-7b. A CCK-8 assay and flow cytometry were used to analyse cell proliferation.

**Results:**

The results showed that H19 was highly expressed in the serum and lungs of MCT-induced rats/mice, and H19 was upregulated by PDGF-BB in vitro. H19 upregulated AT_1_R expression via sponging miRNA let-7b following PDGF-BB stimulation. AT_1_R is a novel target of let-7b. Moreover, the overexpression of H19 and AT_1_R could facilitate PASMCs proliferation in vitro. H19 knockout protected mice from pulmonary artery remodeling and PAH following MCT treatment.

**Conclusion:**

Our study showed that H19 is highly expressed in MCT-induced rodent lungs and upregulated by PDGF-BB. The H19-let-7b-AT_1_R axis contributed to the pathogenesis of PAH by stimulating PASMCs proliferation. The H19 knockout had a protective role in the development of PAH. H19 may be a potential tar-get for the treatment of PAH.

## Background

Pulmonary hypertension (PH) is a complex and fatal disease that is defined as a mean pulmonary arterial pressure (mPAP) over 25 mmHg at rest, as measured by right heart catheterization, and is classified into five groups according to the clinical classification [1, 2]. Pulmonary arterial hypertension (PAH) is recognized as Group 1. The pathological characteristics of PAH are pulmonary vascular remodeling and the right ventricular (RV) hypertrophy, even failure [3]. The pulmonary vascular remodeling is characterized by the excessive proliferation of pulmonary arterial smooth muscle cells (PASMCs) and dysfunction of pulmonary arterial endothelial cells (PAECs), even with thrombosis in situ [4]. The current specific therapies mainly focus on the nitric oxide pathway, the prostacyclin pathway, and the endothelin pathway [5], but the clinical outcomes are not very satisfactory. More studies need to be performed, and novel therapies need to be investigated.

Previous studies suggested that the lungs from PAH patients are characterized by the infiltration of inflammatory cells and an increase in pro-inflammatory cytokines, such as interleukin-1 alpha (IL-1α), interleukin-1 beta (IL-1β), platelet derived growth factors beta polypeptide b (PDGF-BB), interleukin-6 (IL-6), vascular endothelial growth factor (VEGF), and tumor necrosis factor-alpha (TNF-α) [6–9]. Moreover, PDGF-BB promotes PASMCs proliferation [9, 10]. In addition to inflammation, the renin-angiotensin system (RAS) also plays an important role in PAH pathogenesis. The activation of RAS increased the production of peptide hormone angiotensin II (Ang II), which is converted from the inactive pro-hormone angiotensin I (Ang I) to the active peptide hormone Ang II by angiotensin-converting enzyme (ACE) [11]. Ang II enhances PASMCs proliferation and vasoconstriction through the Ang II type 1 receptor (AT_1_R). This pathway is called the ACE-AngII-AT_1_R axis [12, 13]. Although previous studies reported that Losartan and Olmesartan are AT_1_R antagonists and have been used for PAH or hypoxic pulmonary hypertension (HPH) treatment [14–16], they were not very effective. The relationship between inflammatory cytokines and RAS in PAH is not very clear.

Long non-coding RNA (lncRNA) is a type of RNA of more than 200 nucleotides (nt) in length and does not have protein-coding capacity [17]. LncRNAs may contribute to diseases mainly through three mechanisms: chromatin remodeling, transcriptional regulation, and post-transcriptional regulation [18]. Currently, some lncRNAs are reported to be associated with PAH. 362 lncRNAs were reported to be differentially expressed in HPH [19], and 2511 lncRNAs were found to be aberrantly expressed in idiopathic PAH [20]. The lncRNA TCONS_00034812 was identified to modulate pulmonary vascular remodeling via MAPK signaling [21]. The lncRNA MALAT1 may function in PAH pathogenesis [22]. The lncRNA LnRPT repressed PASMCs proliferation by regulating the cell cycle and Notch signaling pathway [10]. The lncRNA H19 is a maternally expressed but paternally imprinted gene in close proximity to chromosome 11p15.5 in the human genome and has a highly conserved secondary structure [23, 24]. H19 is highly expressed in vertebrate embryo development but is downregulated in most adult tissues after birth [25]. It often plays critical roles in the process of tumor metastasis and progression [25, 26]. Furthermore, H19 is related to other important physiological processes, such as hypoxia [27], metabolism [28] and oxidative stress [29]. Additionally, some studies have indicated that H19 may be associated with inflammation. H19 was not only re-expressed in the synovial tissue of rheumatoid arthritis and osteoarthritis but also upregulated by IL-1β, PDGF-BB and TNF-α in vitro [30]. TGF-β1 and IL-6 induced the upregulation of H19, leading to an epithelial-mesenchymal transition (EMT) phenotype in gallbladder cancer [31]. In addition, H19 mainly functions through three patterns: recruiting protein, sponging miRNAs, and the H19/miR-675 axis [32]. H19 harbours the binding sites for miRNA let-7b [33]. H19 may regulate development, cancer progression and cell growth via let-7b [33, 34].

Considering that PAH is inflammation-related and H19 can be regulated by cytokines, we hypothesized that H19 may play an essential role in PAH pathogenesis. This study investigated the role of H19 in PAH and its related mechanism.

## Methods

### Animal treatment

Sprague-Dawley rats (SPF, male, 180–200 g, 4 weeks) and C57/BL6 mice (SPF, male, 25–30 g, 4 weeks, WT mice) were obtained from the Animal Experimental Center of Zhejiang University, China. H19^−/−^ mice (SPF, male, 25–30 g, 4 weeks) were gifts from Luisa Dandolo [35]. Rats received one subcutaneous (sc) injection of 60 mg/kg monocrotaline (MCT) (Sigma Chemicals, St. Louis, MO, USA) and grouped as follows: control (*n* = 6), sc injection of the vehicle saline (0.1 mL/kg); MCT (n = 6), sc injection of MCT [36]. C57/BL6 and H19^−/−^ mice were given a sc injection of MCT at 600 mg/kg once a week for 8 consecutive weeks, and the mice were grouped as above [37]. Rats or mice were isoflurane-anesthetized and sacrificed after 3 or 8 weeks, respectively. Lung and heart tissues were removed, immediately frozen in liquid nitrogen and fixed in 4% buffered paraformaldehyde solution. All experimental procedures were conducted in agreement with the principles approved by the Institutional Animal Care and Use Committee of Zhejiang University.

### Right ventricular systolic pressure (RVSP) and right ventricular hypertrophy measurements

RVSP was measured after MCT treatment in rats and mice. The procedure was conducted as described in previous reports [38, 39]. The right ventricle, left ventricular wall, and ventricular septum were also weighed. The ratio of the right ventricular wall weight to the left ventricular wall plus septum weight [RV/ (LV + S)] was used as an index of right ventricular hypertrophy (*n =* 6 in each group).

### Immunohistochemical analysis

Lung tissues were embedded in paraffin, sectioned at 4-μm thickness and stained with haematoxylin and eosin (H&E). Paraffin-embedded lung sections were stained with α-smooth muscle actin (1:100, ab124964, Abcam, USA). The ratio of pulmonary small artery thickness and muscularization was calculated as described in a previous report [40].

### Isolation of PASMCs and recombinant cytokine treatment

The enzymolysis method was used to isolate rat primary PASMCs as previously described [41]. The cultured cells were trypsinized and seeded in 6-well plates at a density of 5 × 10^5^ cells/ml in DMEM supplemented with 10% fetal bovine serum (FBS) for 24 h. After 24 h, PASMCs were stimulated with recombinant rat IL-1α, IL-1β, IL-6, PDGF-AA, PDGF-BB, TNF-α or VEGF (PeproTech Inc., Rocky Hill, NJ) at 10 ng/ml, 20 ng/ml or 100 ng/ml respectively. After 48 h, the treated cells were washed in sterile PBS and collected for further experiments.

### siRNAs, let-7b mimic, let-7b inhibitor and vector construction

Rno-let-7b-mimic, a miRNA control (miCon), rno-let-7b inhibitor (iLet-7b), an inhibitor control (iCon), siH19, siAT_1_R and siRNA control (siCon) were synthesized by RiboBio (Guangdong, China). The sequences used in this manuscript are shown in Table 1. pGL3-H19, pGL3-H19-Del (binding sequences deleted); pGL3-AT_1_R-3’UTR-WT (wild-type), pGL3-AT_1_R-3’UTR-Mut (binding sequences mutated); pcDNA3.1(−)-H19 (pH 19), pcDNA3.1(−)-AT_1_R (pAT_1_R) and pcDNA3.1(−) (Vector) were synthesized by Genechem (Shanghai, China). The luciferase reporter vectors were constructed by cloning the binding site sequence (or mutant) of H19 or AT_1_R 3’UTR into the pGL3 plasmid. The H19 binding sites were selected according to a previous report [33]. The binding sites of AT_1_R in the 3’UTR were predicted according to the TargetScan and miRDB websites.

**Table 1:** Primers for RT-qPCR

Gene	Accession Number	Species	Primer Pair Sequence 5′ to 3′
H19	NR_027324	Rat	
Sense			GATGACAGGTGTGGTCAACG
Antisense			CAGACATGAGCTGGGTAGCA
H19	NR_130974	Mouse	
Sense			CCACCCACTCACTCAGGATT
Antisense			GGGCAGAAGAGAACTCACCT
AT_1_R	NM_030985	Rat	
Sense			ACCAGGTCAAGTGGATTTCG
Antisense			ATCACCACCAAGCTGTTTCC
AT_1_R	NM_175086	Mouse	
Sense			GCAGCAGGGAGTAACAGAGA
Antisense			TGGGGCAGTCATCTTGGATT
β-actin	NM_031144	Rat	
Sense			TGTCACCAACTGGGACGATA
Antisense			ACCCTCATAGATGGGCACAG
β-actin	NM_007393	Mouse	
Sense			ACTGGGACGACATGGAGAAG
Antisense			ATGGGAGAACGGCAGAAGAA
PCNA	NM_022381	Rat	
Sense			AGGACGGGGTGAAGTTTTCT
Antisense			CAGTGGAGTGGCTTTTGTGA
PCNA	NM_011045	Mouse	
Sense			GAGAGCTTGGCAATGGGAAC
Antisense			ACTCTACAACAAGGGGCACA
Ki67	NM_139186	Rat	
Sense			CATGGGGATTCTGAGGCTAA
Antisense			GGATCACTGCTTGCTCTTCC
Ki67	NM_001081117	Mouse	
Sense			GCCATAACCCGAAAGAGCAG
Antisense			CCAGTTTACGCTTTGCAGGT
5sRNA	NR_033176	Rat	GTCTACGGCCATACCACCCTGAAC
5sRNA	NR_030686	Mouse	CTACAGCACCCGGTATTCCC
Let-7b	NR_031802	Rat	TGAGGTAGTAGGTTGTGTGGTT
Let-7b	NR_029727	Mouse	TGAGGTAGTAGGTTGTGTGGTT
universal r		Rat/Mouse	GCTGTCAACGATACGCTACGTAACG
Anchor RT		Rat/Mouse	GCTGTCAACGATACGCTACGTAACGGCATGACAGTG-ttttttttttttttttttttttttG
Let-7b mimic		Rat	
Sense			UGAGGUAGUAGGUUGUGUGGUU
Antisense			AACCACACAACCUACUACCUCA
Mimic control		Rat	
Sense			UUUGUACUACACAAAAGUACUG
Antisense			CAGUACUUUUGUGUAGUACAAA
Let-7b inhibitor		Rat	AACCACACAACCUACUACCUCA
Inhibitor control		Rat	UUUGUACUACACAAAAGUACUG
si-H19		Rat	
Sense			CCUUUCCUGUAACUCUAUATT
Antisense			UAUAGAGUUACAGGAAAGGTT
si-AT_1_R		Rat	
Sense			GCAAUGUAUGUUUACCUCUAATT
Antisense			UUAGAGGUAAACAUACAUUGCTT
GAPDH	NM_017008	Rat	
Sense			GAGACAGCCGCATCTTCTTG
Antisense			TGACTGTGCCGTTGAACTTG
GAPDH	NM_001289726	Mouse	
Sense			CAACTCCCACTCTTCCACCT
Antisense			GAGTTGGGATAGGGCCTCTC

**Table 2:** Rat primers of cytokines for RT-qPCR

Gene	Accession Number	Species	Primer Pair Sequence 5′ to 3’
IL-1α	NM_017019	Rat	
Sense			TCGGGAGGAGACGACTCTAA
Antisense			GAAAGCTGCGGATGTGAAGT
IL-1β	NM_031512	Rat	
Sense			CTGTGACTCGTGGGATGATG
Antisense			GGGATTTTGTCGTTGCTTGT
PDGF-AA	NM_012801	Rat	
Sense			ATGCCTTGGAGACAAACCTG
Antisense			GTCAAGAAGTTGGCCGATGT
PDGF-BB	NM_031524	Rat	
Sense			ATCGAGCCAAGACACCTCAA
Antisense			ATCACTCCAAGGACCCCATG
IL-6	NM_012589	Rat	
Sense			CTCATTCTGTCTCGAGCCCA
Antisense			CTGTGAAGTCTCCTCTCCGG
TNF-α	NM_012675	Rat	
Sense			CAAACCACCAAGCAGAGGAG
Antisense			GAGGCTGACTTTCTCCTGGT
VEGF	NM_001287107	Rat	
Sense			GCAATGATGAAGCCCTGGAG
Antisense			GCTCTGAACAAGGCTCACAG

### Cells transfection

PASMCs were seeded in a 6-well plate the day before and reached approximately 90% confluence the next day. pH 19, pAT_1_R and vector transfections were performed with 1000 ng of DNA, 5 μl of p3000 in 125 μl of OPTI-MEM and 3.75 μl of lipofectamine 3000 (L3000–015, Invitrogen, NY, USA) in 125 μl of OPTI-MEM. Following 5 min of incubation at room temperature (RT), the DNA mixture and lipofectamine 3000 mixture were mixed by gentle pipetting and incubated for 20 min at RT to allow plasmids/lipid complexes to form. Then, the transfection solution was added, and regular growth medium was added up to 2 ml for each well. The siH19 experiments were performed after stimulating with PDGF-BB with a concentration at 100 ng/ml for 24 h. To prepare the siRNA transfection solution for each well, 50 nM siCon, siH19 or siAT_1_R was mixed with 125 μl of OPTI-MEM by gentle pipetting. Meanwhile, 3.75 μl of lipofectamine 3000 was mixed with 125 μl of OPTI-MEM. The incubation method was described above. After 24 h of incubation at 37 °C in 5% CO_2_, the medium was replaced with fresh growth medium. RNAs and proteins were extracted and analysed at 48 h or the indicated time points following transfection. For the iLet-7b rescue experiments, 50 nM siCon, siH19 or siAT_1_R and 150 nM iCon or iLet-7b was used for each well. For the siAT_1_R rescue experiment, 1000 ng of vector or pH 19 was cotransfected with 50 nM siCon or siAT_1_R for each well.

### Real-time quantitative PCR (RT-qPCR)

Total RNA from lung tissues or PASMCs was extracted with RNA extract reagent (AP-MN-MS-RNA-50, Axygen, USA) according to the manufacturer’s instructions. RNA from the serum was extracted using RNA extract reagent (AP-MN-BL-RNA-50, Axygen, USA). RNA was then reverse transcribed into cDNA using a Prime-Script RT reagent kit (RR047A, Takara, Japan). cDNA was amplified with SYBR Premix Ex Taq™ II (RR820A, Takara, Japan) in LightCycler480 Real-Time PCR system (Applied Biosystems, Foster City, CA) with the following three steps: a hot start at 95 °C for 30 s, followed by 40 cycles of 95 °C for 5 s and 60 °C for 30 s, and a melting curve collected at 95 °C for 5 s and 60 °C for 1 min. The reaction volume was 20 μl containing 1.6 μl of forward or reverse primers (10 μM), 2 μl of cDNA templates, 6.4 μl of ddH_2_O and 10 μl of SYBR. The CT values of the target genes were normalized to 5sRNA (for miRNA let-7b), β-actin (for H19 or mRNA), or GAPDH (for H19 in serum) using the 2^-ΔΔCT^ method, respectively. The S-Poly (T) Plus method was used for miRNA detection as previously described [42]. Primers sequences are listed in Tables 1, 2 and 3.

### Western blotting

Lung tissues or PASMCs were lysed in RIPA buffer containing phosphatase and protease inhibitors (ST505, Beyotime, China) on ice and centrifuged at 12,000 × rpm for 20 min at 4 °C. Protein concentrations were detected using a BCA protein assay kit (Beyotime, China), and equal amounts of protein (40 μg each lane) were subjected to SDS-PAGE and then transferred to PVDF membranes (Millipore, USA). Membranes were incubated by primary antibodies for 18 h, including AT_1_R (1:4000, ab124734, Abcam, USA) and α-tubulin (1:5000, T5168; Sigma, St. Louis, MO, USA), followed by HRP-conjugated secondary antibodies for 2 h at room temperature. ECL was used to detect the immunoreactive bands, and blots densitometry was analysed by ImageJ software.

### Dual luciferase reporter assay

HEK-293 T cells were seeded in 24-well plates at a density of 1 × 10^4^ cells/ml. After 24 h, HEK-293 T cells were cotransfected with 200 ng of target plasmids and 20 ng of Renilla plasmid along with 40 nM miCon or 10/20/40 nM let-7b mimic using 1.5 μl of lipofectamine 3000 for each well. The luciferase activities were measured 48 h after transfection using a dual-luciferase assay kit (E2920, Promega, USA).

### CCK-8 assay, cell cycle analysis and scratch wound healing assay

A cell counting kit-8 (CCK-8) assay (CK04, Dojindo, Kumamoto, Japan) was used to detect the proliferation of PASMCs. PASMCs were seeded in 96-wells plates at a concentration of 1 × 10^4^ cells/well. After treatment for 6 h, 12 h, 24 h, 48 h, and 72 h, PASMCs were washed with sterile PBS and WST-8 [2-(2-methoxy-4-nitrophenyl)-3-(4-nitrophenyl)-5-(2,4-disulfophenyl)-2H-tetrazolium, monosodium salt] was added to each well for 2 h. Then, the optical density value of absorbance at 450 nm was measured. Cell cycle analysis was measured by flow cytometry as described previously [38]. In brief, treated cells were collected and fixed in precooled 75% alcohol at 4 °C overnight. The fixed cells were then washed with precooled sterile PBS three times and stained using a cell-cycle staining kit (550,825, Biosciences, USA), which contained 0.1 mg/ml RNase, for 15 min in the dark at RT,. The stained cells were sorted by FACS Calibur (BD Biosciences, NJ, USA), and cell cycle distribution was determined using ModFit LT software (Verity Software House, Topsham, ME, USA).

A scratch wound healing assay was conducted as follows: PASMCs were cultured in 24-well plates for 24 h in DMEM supplemented with 10% FBS until the cells reached 80–90% confluency. Linear wound tracks were generated with sterile, 1 ml pipettes and maintained under standard conditions. The scratched cells were rinsed twice with sterile PBS to remove non-adherent cells, and 0.2% FBS in DMEM was added. Photographs of the centers of the gaps were taken using a 40× phase-contrast microscope (Zeiss). Cell migration at 0 and 24 h after scratching was evaluated by determining the wound distance at two random wound gap locations.

### Statistical analysis

Quantitative data were presented as the mean ± standard deviation (SD). Student’s *t*-test was selected to compare the difference between the two groups. One-way or two-way ANOVA followed by post hoc Tukey’s test was used to compare the difference among three or more groups. All statistical analyses were performed with SPSS 19.0 (Chicago, IL, USA) and GraphPad Prism 5 software (La Jolla, CA). In figure legends, n refers to number of samples. All experimental groups contain at least three biological replicates. *P* < 0.05 was considered to be statistically significant.

## Results

### H19 is upregulated in rat lungs following MCT treatment and is induced by cytokines in vitro

**Fig. 1:**
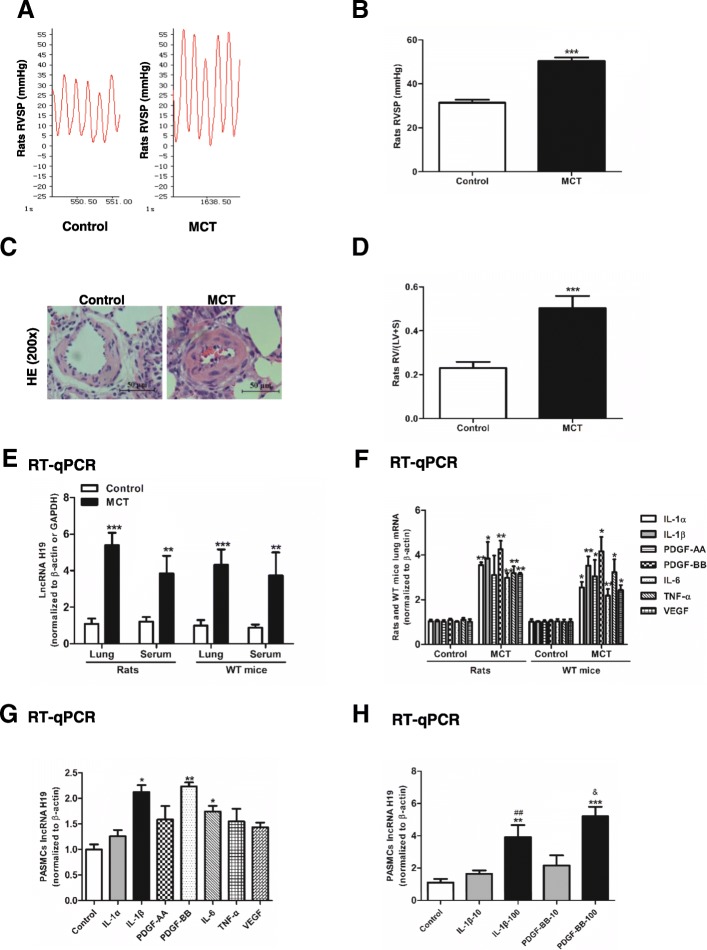
H19 is upregulated in rat lungs following MCT treatment and is induced by cytokines in vitro. **a**: At 3 weeks after injecting rats with MCT (60 mg/kg) or saline (control), the right ventricular systolic pressure (RVSP) was measured. **b**: Summary of representative RVSP measurements. **c**: Representative microphotographs of pulmonary small arteries. Lung sections stained with haematoxylin-Eosin (H&E). Scale bar = 50 μm. **d**: The ratio of right ventricle to left ventricle plus septum weight RV/ (LV + S). **e**: Expression of H19 in the lung or serum of MCT-induced rats and C57/BL6 mice (WT mice). **f**: Expression of IL-1α, IL-1β, IL-6, PDGF-AA, PDGF-BB, TNF-α and VEGF in the lungs of MCT-induced rats and WT mice. **g**: Expression of H19 in PASMCs stimulated by cytokines at a concentration of 20 ng/ml for 48 h. **h**: Expression of H19 in PASMCs stimulated with IL-1β and PDGF-BB at a concentration of 10 and 100 ng/ml for 48 h. Values were presented as means ± SD (*n* = 6 in each group). *0.01 ≤ *P* ≤ 0.05 (different from the corresponding control); **0.001 ≤ *P* ≤ 0.009 (different from the corresponding control); ***P < 0.001 (different from the corresponding control); ##0.001 ≤ *P* ≤ 0.009 (IL-1β-100 vs. IL-1β-10); &0.001 ≤ *P* ≤ 0.009 (PDGF-BB-100 vs. PDGF-BB-10)

The MCT-induced PAH model was successfully established according to a previous report [36]. Rats injected with 60 mg/kg MCT displayed significantly pulmonary artery remolding and PH with the mean RVSP elevating to 50.40 ± 1.59 mmHg compared with 31.40 ± 1.37 mmHg in the control (*P* < 0.001, Fig. 1a, b and c). RV hypertrophy was indicated by the increase in the ratio of RV/ (LV + S) compared with the control (0.50 ± 0.56 in MCT group vs. 0.24 ± 0.03 in control group, *P* < 0.001, Fig. 1d). To explore whether the expression of H19 changed in MCT-induced rats lungs, a lung homogenate was used for RT-qPCR. The expression of H19 was increased 5.00-fold in MCT-induced rat lungs (*P* < 0.001) and 4.34-fold in MCT-induced WT mouse lungs compared to controls (*P* < 0.001, Fig. 1e). H19 was also upregulated in the serum of MCT-induced rats and MCT-induced WT mice (*P* = 0.003 and *P* = 0.003, respectively, Fig. 1e). To test whether inflammation affected the expression of H19 in vitro, seven upregulated cytokines were selected to stimulate PASMCs (Fig. 1f), including IL-1α, IL-1β, PDGF-AA, PDGF-BB, IL-6, TNF-α, and VEGF. Among the cytokines, IL-1β and PDGF-BB resulted in the strongest stimuli of H19 (2.1-fold in IL-1β group and 2.2-fold in PDGF-BB group compared to controls; *P* = 0.016 and *P* = 0.002, respectively, Fig. 1g). The expression of H19 exhibited a dose-dependent effect when the PASMCs were stimulated by different doses of IL-1β and PDGF-BB (Fig. 1h).

**Table 3:** Mouse primers of cytokines for RT-qPCR

Gene	Accession Number	Species	Primer Pair Sequence 5′ to 3′
IL-1α	NM_010554	Mouse	
Sense			AGCAGCCTTATTTCGGGAGT
Antisense			ATCATATGTCGGGGTGGCTC
IL-1β	NM_008361	Mouse	
Sense			GGCTCATCTGGGATCCTCTC
Antisense			TCATCTTTTGGGGTCCGTCA
PDGF-AA	NR_156469	Mouse	
Sense			GATCCACCTCGCATCATCTT
Antisense			GTTCCCGACAGGAAAACTCA
PDGF-BB	NM_011057	Mouse	
Sense			GATCTCTCGGAACCTCATCG
Antisense			GGCTTCTTTCGCACAATCTC
IL-6	NM_031168	Mouse	
Sense			CTGGGGATGTCTGTAGCTCA
Antisense			CTGTGAAGTCTCCTCTCCGG
TNF-α	NM_013693	Mouse	
Sense			GACCCCTTTACTCTGACCCC
Antisense			AGGCTCCAGTGAATTCGGAA
VEGF	NM_001287056	Mouse	
Sense			GCTGTAACGATGAAGCCCTG
Antisense			TCGTCTTCTCACCCTCAACC

### H19 interacts with miRNA let-7b

**Fig. 2:**
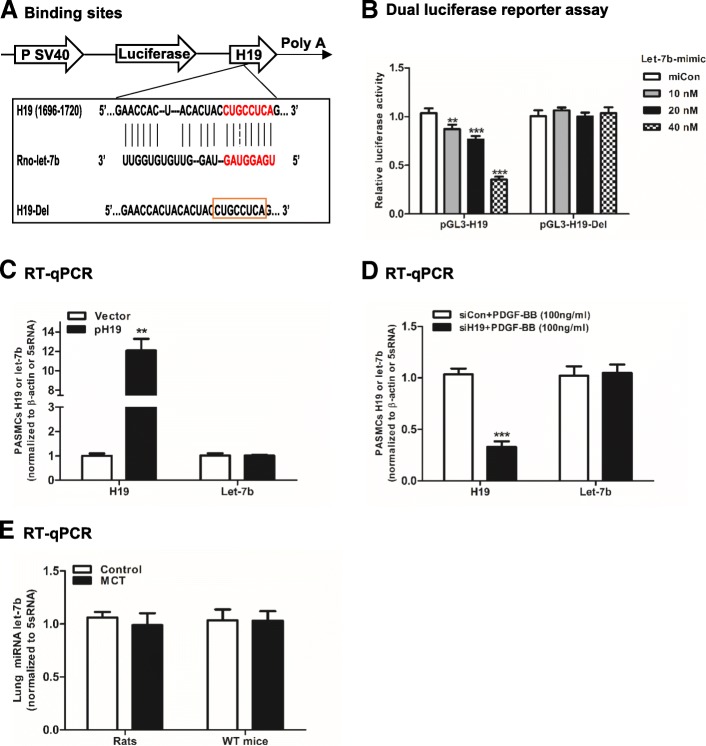
H19 interacts with miRNA let-7b. **a**: The diagram shows the structure of H19 luciferase in pGL3 reporter. The predicted binding sites for sponging let-7b and the corresponding deletion sites in the H19 sequence. The H19-Del represented the binding sites deletion (noted with a square red box). **b**: Dual luciferase activity assay. HEK-293 T cells were cotransfected with 200 ng of pGL3-H19 or pGL3-H19-Del with 20 ng of Renilla plasmid. A 40 nM miRNA control (miCon) or let-7b-mimic at a concentration of 10 nM, 20 nM and 40 nM was also added. **c**: PASMCs were transfected with 1000 ng of vector or pH 19 for 48 h. **d**: PASMCs were stimulated with PDGF-BB at a concentration of 100 ng/ml for 48 h. Then, PASMCs were transfected with 50 nM siRNA control (siCon) or siH19 for 24 h. **e**: Expression of let-7b in the lungs of MCT-induced rats and C57/BL6 mice (WT mice). H19 and let-7b were detected by RT-qPCR and normalized to β-actin and 5sRNA, respectively. Values were presented as means ± SD from 3 independent experiments. **0.001 ≤ *P* ≤ 0.009 (different from the corresponding control); ***P < 0.001 (different from the corresponding control)

According to a previous report [33], the binding sites for sponging let-7b and the corresponding deletion sites in H19 were designed (Fig. 2a). The luciferase activity of pGL3-H19 decreased in a dose dependent manner when the cells were cotransfected with a let-7b mimic. In contrast, the luciferase activity was unchanged when the pGL3-H19-Del and let-7b mimic were cotransfected into cells (Fig. 2b). As shown in Fig. 2c and d, the expression of let-7b was not influenced if H19 was overexpressed or knocked down. Moreover, the expression of let-7b was not influenced in the MCT-induced PAH rodent models (Fig. 2e). These results showed that H19 may only sequester let-7b by binding certain sites rather than degrading it.

### AT_1_R is a novel target of let-7b

**Fig. 3:**
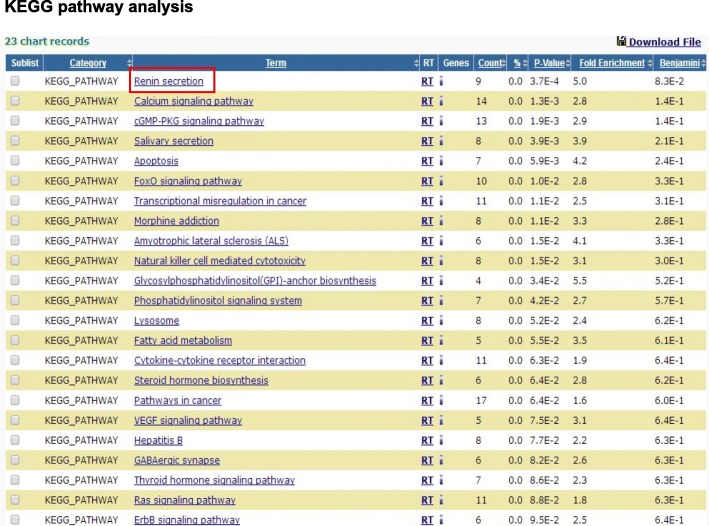
KEGG pathway analysis. The results of the KEGG pathway analysis. The target genes of let-7b selected from TargetScan were evaluated using the KEGG pathway analysis

**Fig. 4:**
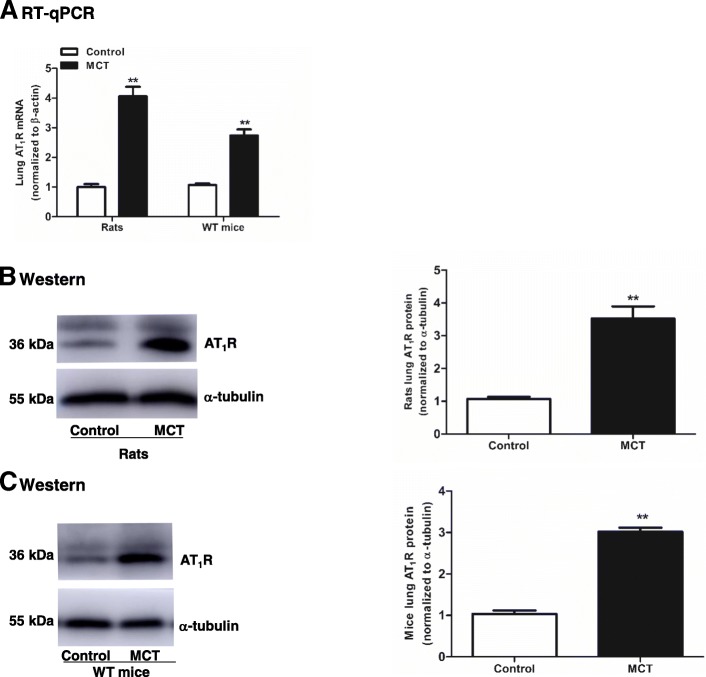
AT_1_R is upregulated in the lungs of MCT-induced rats and WT mice. Rats and WT mice were treated with MCT. The mRNA (**a**) and protein (**b**, **c**) of AT_1_R in the lungs of rats and WT mice were detected. Values were presented as means ± SD (n = 6 in each group). **0.001 ≤ *P* ≤ 0.009 (different from the corresponding control)

The TargetScan website was used to analyse the potential targets of let-7b, and then these target genes were evaluated by KEGG analysis. The pathway results showed that let-7b may regulate several genes in RAS (Fig. 3). Among these genes, AT_1_R was selected because it was significantly upregulated in the lungs of MCT-induced rats and WT mice (Fig. 4).

**Fig. 5:**
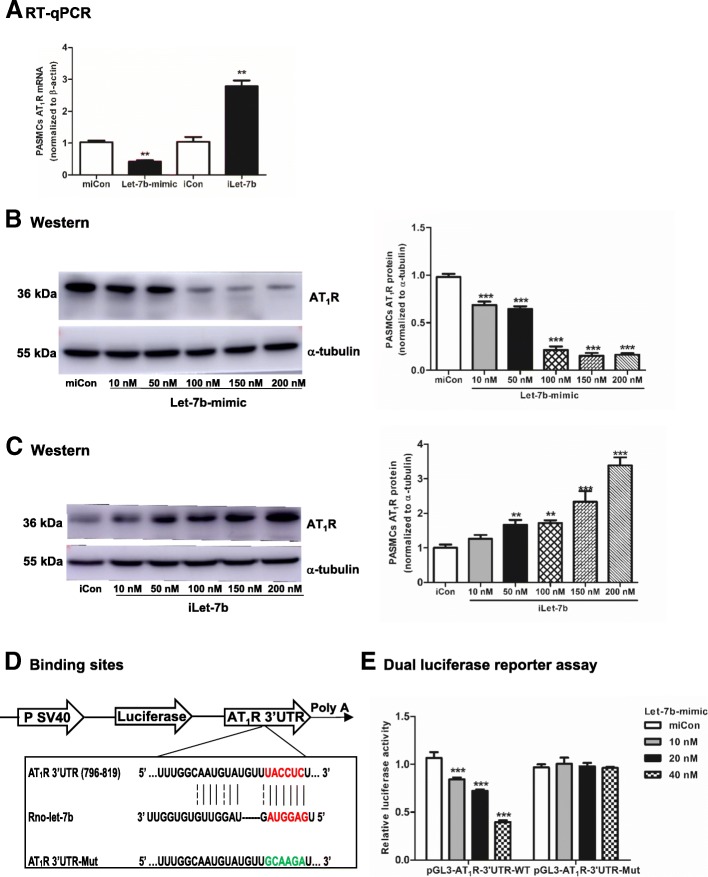
AT_1_R is a novel target of miRNA let-7b. **a**: PASMCs were transfected with 50 nM miCon, let-7b-mimic, iCon or iLet-7b for 48 h. AT_1_R mRNA was detected by RT-qPCR. **b**: PASMCs were transfected with 50 nM miCon or let-7b-mimic at a concentration of 10 nM, 50 nM, 100 nM, 150 nM or 200 nM for 48 h. **c**: PASMCs were transfected with 50 nM iCon or iLet-7b at a concentration of 10 nM, 50 nM, 100 nM, 150 nM or 200 nM for 48 h. AT_1_R protein was detected by Western Blot. **d**: The diagram shows the structure of AT_1_R 3’UTR luciferase in pGL3 reporter. The binding sites of let-7b in the AT_1_R 3’UTR sequence and the corresponding mutant sites (shown in green). **e**: Dual luciferase reporter assay. HEK-293 T cells were cotransfected with 200 ng of pGL3-AT_1_R-3’UTR-WT or pGL3-AT_1_R-3’UTR-Mut and 20 ng of Renilla plasmid. miCon or let-7b-mimic at a concentration of 10 nM, 20 nM and 40 nM was also added. Values were presented as means ± SD from 3 independent experiments. **0.001 ≤ *P* ≤ 0.009 (different from the corresponding control); ***P < 0.001 (different from the corresponding control)

AT_1_R mRNA was decreased by 2.45-fold when the let-7b mimic was transfected and increased by 2.67-fold when iLet-7b was transfected (Fig. 5a). The AT_1_R protein level gradually decreased with higher concentrations of let-7b mimic and increased with the higher concentrations of iLet-7b (Fig. 5b, c).The binding sites for let-7b in the AT_1_R 3’UTR sequence were searched using the miRDB website. The corresponding mutant sites in the AT_1_R 3’UTR sequence were indicated (Fig. 5d). Furthermore, the luciferase activity of pGL3-AT_1_R-3’UTR-WT was decreased in a dose-dependent manner when the let-7b mimic was cotransfected. In contrast, the luciferase activity was unchanged when pGL3-AT_1_R-3’UTR-Mut and the let-7b mimic were cotransfected into cells (Fig. 5e). These results indicated that AT_1_R is a novel target of let-7b.

### PDGF-BB-H19-let-7b axis regulates AT_1_R expression

**Fig. 6:**
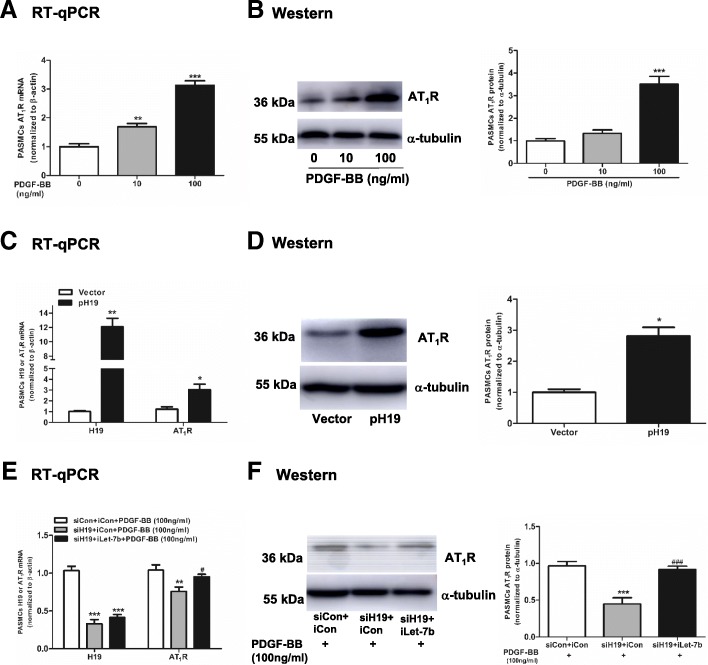
PDGF-BB-H19-Let-7b axis regulates AT_1_R expression. PASMCs were stimulated with PDGF-BB at a concentration of 0, 10 and 100 ng/ml for 48 h. AT_1_R mRNA (**a**) and protein (**b**) were detected by RT-qPCR and Western Blot, respectively. PASMCs were transfected with 1000 ng of vector or pH 19 for 48 h. H19 and AT_1_R mRNA (**c**) were detected by RT-qPCR, and AT_1_R protein (**d**) was detected by Western Blot. PASMCs were stimulated with PDGF-BB at a concentration of 100 ng/ml for 48 h. Then, PASMCs were transfected with (50 nM siCon+ 150 nM iCon), (50 nM siH19 + 150 nM iCon) or (50 nM siH19 + 150 nM iLet-7b) for 24 h, respectively. H19 and AT_1_R mRNA (**e**) were detected by RT-qPCR, and AT_1_R protein (**f**) was detected by Western Blot. Values were presented as means ± SD from 3 independent experiments. *0.01 ≤ *P* ≤ 0.05 (different from the corresponding control); **0.001 ≤ *P* ≤ 0.009 (different from the corresponding control); ***P < 0.001 (different from the corresponding control); #0.01 ≤ *P* ≤ 0.05 [(siH19 + iLet-7b) vs. (siH19 + iCon)]; ###*P* < 0.001 [(siH19 + iLet-7b) vs. (siH19 + iCon)]

AT_1_R was significantly upregulated by PDGF-BB in a dose-dependent manner (Fig. 6a, b). AT_1_R abundance was increased when H19 was overexpressed at both the mRNA and protein levels (*P* = 0.044 for mRNA and *P* = 0.015 for protein, Fig. 6c, d). Furthermore, after PDGF-BB stimulation, AT_1_R expression was repressed at both the mRNA and protein levels when H19 was knocked down (*P* = 0.002 for mRNA and *P* < 0.001 for protein), but this effect was attenuated by iLet-7b transfection (Fig. 6e, f). These results demonstrated that AT_1_R is regulated by the PDGF-BB-H19-Let-7b axis.

### PDGF-BB-H19-let-7b axis promotes the proliferation of PASMCs through AT_1_R signaling

**Fig. 7:**
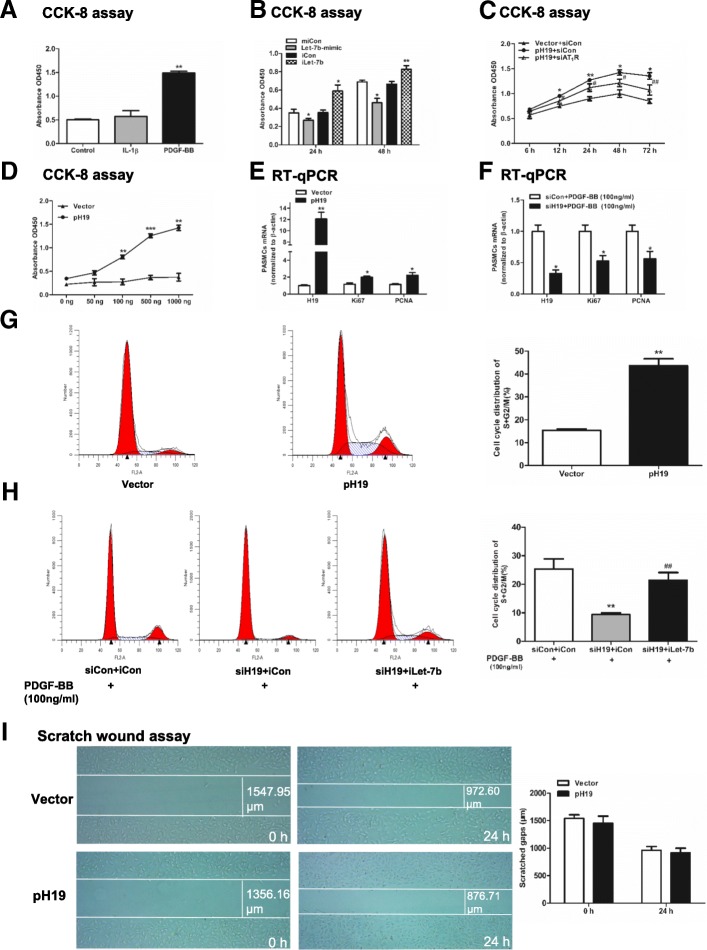
PDGF-BB-H19-Let-7b axis promotes the proliferation of PASMCs. **a**: PASMCs were stimulated with IL-1β and PDGF-BB at a concentration of 100 ng/ml for 48 h. **b**: PASMCs were transfected with 50 nM miCon, let-7b-mimic, iCon or iLet-7b for 24 h and 48 h. **c**: PASMCs were cotransfected with 1000 ng of vector or pH 19 with 50 nM siCon or siAT_1_R for 6 h, 12 h, 24 h, 48 h and 72 h, respectively. **d**: PASMCs were transfected with vector or pH 19 at 0 ng, 50 ng, 100 ng, 500 ng and 1000 ng for 48 h. In **a**, **b**, **c** and **d**, PASMCs were washed with sterile PBS after transfection or stimulation and cultured in CCK8 diluent for 2 h. Absorbance was detected at 450 mm. **e**: PASMCs were transfected with 1000 ng of vector or pH 19 for 48 h. **f**: PASMCs were stimulated with PDGF-BB at a concentration of 100 ng/ml for 48 h. Then, PASMCs were transfected with 50 nM siCon or siH19 for 24 h. H19, Ki67 and PCNA mRNA were detected by RT-qPCR. **g**: PASMCs were transfected with 1000 ng of vector or pH 19 for 48 h. **h**: PASMCs were stimulated with PDGF-BB at a concentration of 100 ng/ml for 48 h. Then, PASMCs were transfected with (50 nM siCon + 150 nM iCon), (50 nM siH19 + 150 nM iCon) or (50 nM siH19 + 150 nM iLet-7b) for 24 h. Cell-cycle distribution was analysed by flow cytometry. **i**: Scratch wound healing assay of PASMCs transfected with 1000 ng of vector or pH 19. Values were presented as means ± SD from 3 independent experiments. *0.01 ≤ P ≤ 0.05 (different from the corresponding control); **0.001 ≤ *P* ≤ 0.009 (different from the corresponding control); ***P < 0.001 (different from the corresponding control); #0.01 ≤ P ≤ 0.05 [different from the (pH 19 + siCon) group]; ##0.001 ≤ *P* ≤ 0.009 [(siH19 + iLet-7b) vs. (siH19 + iCon) or (pH 19 + siCon) vs. (pH 19 + siAT_1_R)]

PASMCs proliferation significantly increased when the cells were stimulated by PDGF-BB (*P* = 0.001), while it showed no alteration with IL-1β stimulation (Fig. 7a). Moreover, the let-7b mimic significantly decreased the proliferation of PASMCs (*P* = 0.019 at 24 h and *P* = 0.028 at 48 h), while iLet-7b displayed the opposite effect (*P* = 0.047 at 24 h and P = 0.002 at 48 h) (Fig. 7b). To clarify whether PDGF-BB induced the proliferation of PASMCs via H19, H19 was overexpressed at different time points or with different plasmid doses in PASMCs. As shown in Fig. 7c, d, the absorbance at 450 mm was gradually increased in a H19 dose- and time-dependent manner. Moreover, this pro-proliferation effect could be reversed by siAT_1_R. The mRNA levels of the proliferation makers Ki67 and PCNA were upregulated when H19 was overexpressed (*P* = 0.023, 0.033, respectively) and downregulated with siH19 transfection after PDGF-BB stimulation (*P* = 0.017, 0.010, respectively, Fig. 7e, f). When H19 was overexpressed, the number of PASMCs in the S + G2/M phase increased from 15.45 ± 0.58% to 43.66 ± 2.99% in the cell cycle assay (*P* = 0.003, Fig. 7g). However, after PDGF-BB stimulation, the number of PASMCs in the S + G2/M phase was decreased by siH19 transfection (*P* = 0.001), and this effect was rescued by iLet-7b transfection (*P* = 0.003, siH19 + iLet-7b vs. siH19 + iCon, Fig. 7h). However, the migration ability of PASMCs was unchanged when H19 was overexpressed (Fig. 7i). These results indicated that H19 regulates the proliferation of PASMCs via sponging let-7b after treatment with PDGF-BB.

**Fig. 8:**
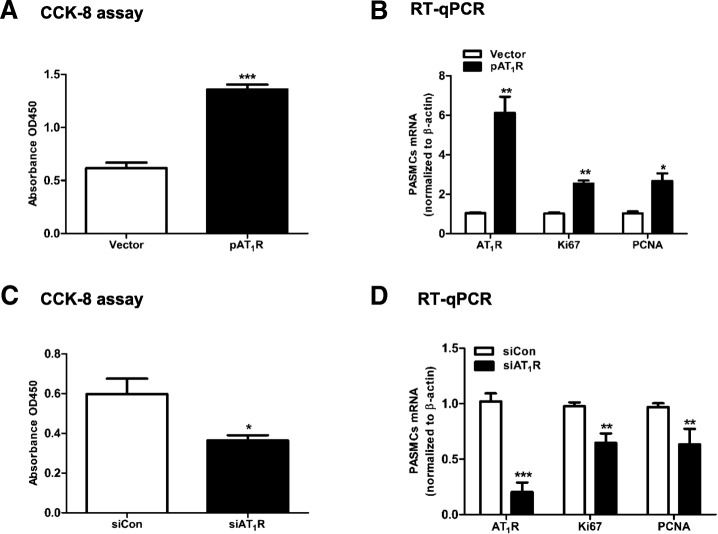
AT_1_R promotes the proliferation of PASMCs. PASMCs were transfected with 1000 ng of vector or pAT_1_R for 48 h (**a**, **b**). PASMCs were also transfected with 50 nM siCon or siAT_1_R for 48 h (**c**, **d**). Proliferation was analyzed by CCK-8 assay. AT_1_R, Ki67 and PCNA mRNA were detected by RT-qPCR. Values were presented as means ± SD from 3 independent experiments. *0.01 ≤ P ≤ 0.05 (different from the corresponding control); **0.001 ≤ *P* ≤ 0.009 (different from the corresponding control); ***P < 0.001 (different from the corresponding control)

In addition, the absorbance at 450 mm was increased when AT_1_R was overexpressed (*P* < 0.001, Fig. 8a). PCNA and Ki67 mRNA were also upregulated (Fig. 8b). In contrast, the absorbance at 450 mm was decreased when AT_1_R was knocked down (Fig. 8c). Similarly, PCNA and Ki67 mRNAs were downregulated (Fig. 8d). These results demonstrated that the PDGF-BB-H19-Let-7b axis promotes PASMCs proliferation through AT_1_R.

### H19 knockout protects mice from pulmonary artery remodeling and PAH

**Fig. 9:**
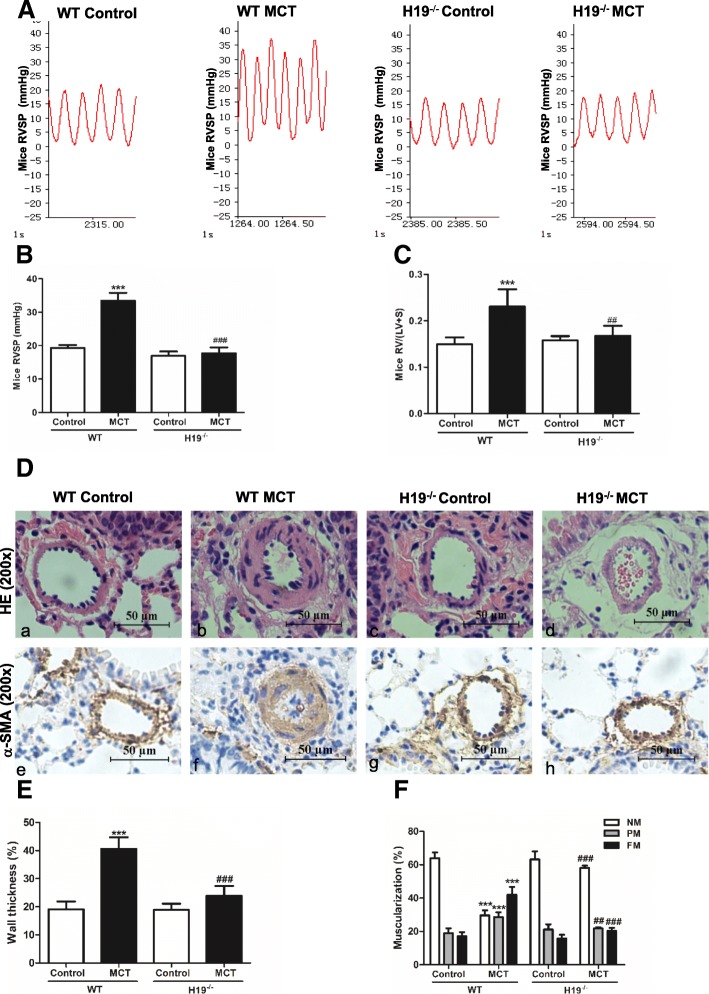
H19 knockout protects mice from pulmonary artery remodeling and PAH. After WT and H19^−/−^ mice were injected with saline or MCT (600 mg/kg) continuously for 8 weeks, RVSP was detected (**a**, **b**). **c**: The ratio of RV/ (LV + S). **d**: H&E staining and immunohistochemical staining of α-smooth muscle actin were performed in the lung sections. Representative images of pulmonary small arteries. Scale bar = 50 μm. Quantification of wall thickness (**e**) and vessel muscularization (**f**). Values were presented as means ± SD (*n* = 6 in each group). Only vessels with diameter between 30 and 90 μm were analyzed. NM, nonmuscularized vessels; PM, partially muscularized vessels; FM, fully muscularized vessels. ***P < 0.001 (different from the corresponding control). ##0.001 ≤ *P* ≤ 0.009 [RV/ (LV + S) or PM in H19^−/−^-MCT group vs. WT-MCT group]; ###*P* < 0.001 [RVSP, wall thickness (%), NM or FM in H19^−/−^-MCT group vs. WT-MCT group]

To explore the role of H19 in vivo, H19^−/−^ mice were used. The mean RVSP was significantly elevated in WT MCT mice compared with the control group (33.41 ± 2.40 mmHg vs. 19.32 ± 0.87 mmHg, *P* < 0.001). However, the mean RVSP in H19^−/−^ MCT mice was not higher than that in the H19^−/−^ control group (Fig. 9a, b). Additionally, the ratio of RV/ (LV + S) was increased by approximately 37.5% in the WT MCT mice (0.15 ± 0.01 in the WT control group vs. 0.23 ± 0.04 in the WT MCT group, *P* < 0.001). However, the ratio of RV/ (LV + S) in the H19^−/−^ MCT mice was not significantly higher than that in the control group (Fig. 9c). H&E staining and immunostaining with α-smooth muscle actin indicated that the medial walls of the pulmonary small arteries were markedly thickened in the WT MCT mice group. This effect was attenuated by H19 gene knockout (Fig. 9d, e). Moreover, in WT mice control lungs, 63.98 ± 3.38% of the arterioles were non-muscularized vessels, and 17.13 ± 2.47% were fully muscularized vessels. In contrast, fully muscularized vessels occupied a greater proportion (41.91 ± 4.76%) in the WT MCT mice, while non-muscularized vessels showed a lower proportion (29.55 ± 3.11%). The H19 gene knockout mainly increased the percentage of non-muscularized vessels (58.05 ± 1.38%) and reduced the percentage of fully muscularized vessels (20.22 ± 1.97%) compared to the WT MCT mice (*P* < 0.001 in NM and *P* < 0.001 in FM, Fig. 9f).

**Fig. 10:**
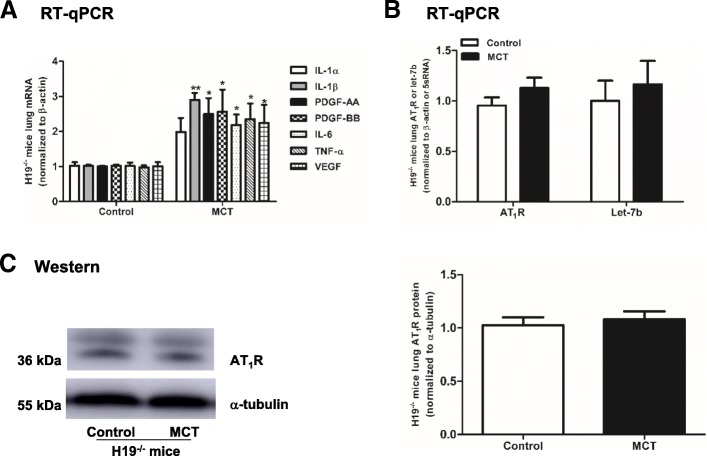
Cytokines, let-7b and AT_1_R expression in the lungs of H19^−/−^ mice treated with MCT. **a**: Expression of IL-1α, IL-1β, IL-6, PDGF-AA, PDGF-BB, TNF-α and VEGF mRNA in the lungs of H19^−/−^ mice. **b**: Expression of AT_1_R mRNA and let-7b in the lungs of H19^−/−^ mice. **c**: Protein level of AT_1_R in the lungs of H19^−/−^ mice. Cytokines, let-7b and AT_1_R mRNA were detected by RT-qPCR, and AT_1_R protein was detected by Western Blot. Values were presented as means ± SD (n = 6 in each group). *0.01 ≤ *P* ≤ 0.05(different from the corresponding control); **0.001 ≤ *P* ≤ 0.009(different from the corresponding control

Next, cytokines in the lungs of H19^−/−^ mice were analysed. IL-1β was the most increased cytokine in the H19^−/−^ MCT group by 2.84-fold compared with the H19^−/−^ control group (*P* = 0.005, Fig. 10a). The levels of let-7b and AT_1_R displayed no significant changes in the H19^−/−^ MCT group (Fig. 10b, c).

## Discussion

The main objective of this study was to determine whether H19 contributes to the pathogenesis of PAH. Our results showed that H19 was highly expressed in MCT-induced rodent lungs and upregulated by PDGF-BB in vitro. H19 upregulated AT_1_R expression via sponging let-7b following PDGF-BB stimulation. AT_1_R is a novel target of let-7b. Moreover, overexpression of H19 and AT_1_R could facilitate PASMCs proliferation in vitro. H19 knockout protected mice from pulmonary artery remodeling and PAH following MCT treatment.

LncRNA H19 was one of the first imprinted lncRNAs to be discovered and is regarded as an oncofetal or tumour-suppressed gene [43]. H19 plays essential roles in tumor proliferation, invasion, apoptosis and angiogenesis [24]. H19 has been reported to be upregulated and promote cell proliferation in breast cancer [44], gastric cancer [45, 46], glioblastoma [47] and lung cancer [26], while it is downregulated and decreases proliferation in papillary thyroid carcinoma [48] and hepatocellular carcinoma [49]. This study is the first to find that H19 contributes to the pathogenesis of PAH mediated by AT_1_R. AT_1_R belongs to the seven transmembrane domain, G protein-coupled receptor (GPCR) superfamily. AngII binding to AT_1_R contributes to the coupling of G proteins (Gq/G11 and/or Gi/Go) to the C-terminal of AT_1_R and thus activates many intracellular signaling pathways, including MAPK signaling [12]. AT_1_R is an important component of RAS. Previous reports showed that the ACE-AngII-AT_1_R axis contributes to the vasoconstriction and proliferation of pulmonary arteries [11, 50]. AT_1_R plays an important role in PAH by enhancing vascular proliferation through the activation of MAPK and RhoA signaling [12].

**Fig. 11:**
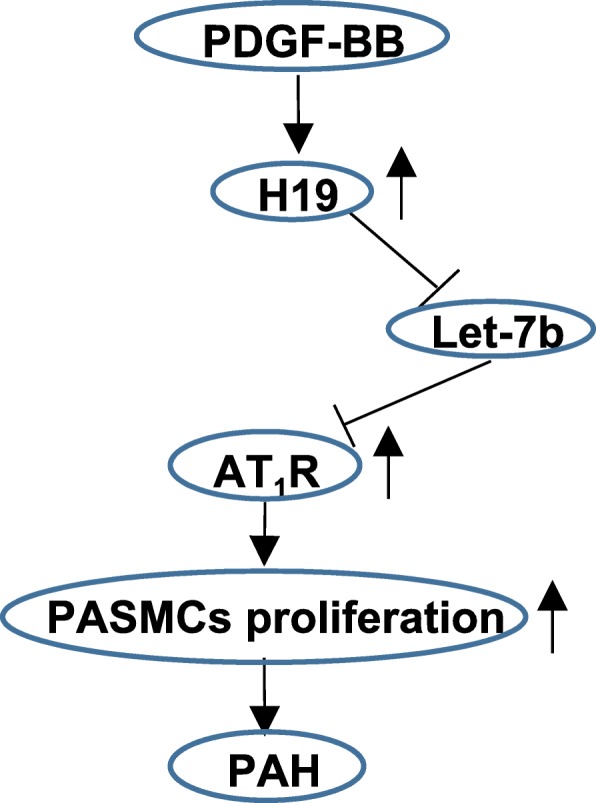
Scheme of the regulation of PDGF-BB-H19-Let-7b axis to PASMCs proliferation in PAH

Consist with a previous report [33], our results suggest that H19 sponges let-7b. MiRNAs are small RNAs with sequences ranging from 21 to 23 nt and that induce the degradation or inhibit the translation of target mRNAs via RNA interference [51]. Let-7 family members include let-7a to 7i, miR-98 and miR-202, and are involved in multiple human cancers [52]. Let-7 promotes cell growth via targeting the IGF1 receptor (IGF1R) and cyclin D1 [34, 53, 54]. Let-7 family members also participate in PH. A previous study reported that let-7 g inhibited PASMCs proliferation in HPH [55]. Moreover, the reduced expression of let-7b increased the expression of ET-1 and thus affected PASMCs and PAECs proliferation in chronic thromboembolic pulmonary hypertension [56]. Our results were the first to confirm that AT_1_R is a novel target of let-7b and that the H19-let-7b-AT_1_R axis contributes to the pathogenesis of PAH by stimulating PASMCs proliferation (Fig. 11).

Many cytokines participate in the pathogenesis of PAH. Consist with previous reports, we found that IL-1α, IL-1β, PDGF-AA, PDGF-BB, IL-6, TNF-α, and VEGF were upregulated in rat and mouse lungs following MCT treatment. How these cytokines lead to PAH remained elusive. Similar to previous studies [9], our results showed that PDGF-BB activated the proliferation of PASMCs. The relationship between inflammatory cytokines and H19 is controversial. Some researchers have reported that H19 is re-expressed or overexpressed when stimulated by TGF-β1, IL-6, IL-1β, PDGF-BB and TNF-α [30, 31]. On the contrary, other reports have shown that H19 is downregulated by IL-1β, TNF-α, IFN-γ, IL-4, IL-6, IL-17A and IL-22 [57, 58]. No consistent conclusion has been established yet, perhaps because of the diversity of pathological processes and different types of cells and tissues. Our results demonstrated that H19 was significantly upregulated in PASMCs stimulated by PDGF-BB and IL-1β. However, the proliferation of PASMCs was not significantly enhanced by IL-1β stimulation. It is controversial that IL-1β displays a pro-proliferative effect on PASMCs. Parpaleix A et al. reported that IL-1β treatment stimulated PASMCs growth [59]. In contrast, Akihide et al. reported that the proliferation of PASMCs was not significantly affected by IL-1β [60]. The IL-1 family members are considered to be ‘early-response’ cytokines, release in the earliest stage of an immune response and trigger proinflammatory cytokines releasing [61]. Therefore, we hypothesized that IL-1β may upregulate the expression of H19 through a different pathway and affect other functions of PASMCs. However, this proposal needs further validation.

A detailed mechanism remains unclear. As previously reported, H19 was minimally detected in most adult tissues but is markedly re-expressed after vascular injury, probably because H19 is associated with vascular smooth muscle cells differentiation [62]. The injured pulmonary small arteries in PAH may lead to upregulation of H19. However, this hypothesis needs to be further validated.

We found that the concentrations of H19 in the serum of MCT-induced rats and WT mice were higher than those of the controls. The elevated H19 in the serum may be released from lung tissues. LncRNAs have been applied as fluid-based markers in cancers. For example, the lncRNA DD3 and lncRNA HULC have been used as diagnostic markers for patients with prostate cancer and hepatocarcinoma, respectively [25]. Moreover, H19 was detected in blood samples from atherosclerotic patients as previously reported [63]. Therefore, we suggested that H19 may be utilized as a potential diagnosis marker for PAH.

However, there are some limitations in this study. First, we did not explore the expression of H19 in the serum and lung tissue of PAH patients. Would serum H19 be a diagnostic or prognostic marker for PAH patients? Second, the detailed mechanism by which PDGF-BB regulates H19 is unclear. Further clinical experiments are required to determine whether H19 can be a therapeutic target for PAH or HPH. Further studies are needed to answer the above problems.

## Conclusion

In conclusion, our study showed that H19 was highly expressed in MCT-induced rodent lungs and upregulated by PDGF-BB. H19 upregulated AT_1_R expression via sponging miRNA let-7b, and AT_1_R is a novel target of let-7b. The H19-let-7b-AT_1_R axis contributed to the pathogenesis of PAH by stimulating PASMCs proliferation. H19 knockout protected mice from pulmonary artery remodeling and PAH following MCT treatment. H19 maybe a potential target for the treatment of PAH, and more research is necessary to validate this possibility.

## Abbreviations

ACE: Angiotensin-converting enzyme
Ang I: Angiotensin I
Ang II: Angiotensin II
AT_1_R: Ang II type 1 receptor
EMT: Epithelial-mesenchymal transition
FBS: Fetal bovine serum
HPH: Hypoxic pulmonary hypertension
iCon: Inhibitor control; siCon, siRNA control
IL-1α: Interleukin-1 alpha
IL-1β: Interleukin-1 beta
IL-6: Interleukin-6
iLet-7b: Rno-let-7b inhibitor
lncRNA: Long non-coding RNA
MCT: Monocrotaline
miCon: miRNA control
PAECs: Pulmonary arterial endothelial cells
PAH: Pulmonary arterial hypertension
PASMCs: Pulmonary arterial smooth muscle cells
PDGF-BB: Platelet derived growth factors beta polypeptide b
RAS: Renin-angiotensin system
RV: Right ventricular
RV/ (LV + S): The ratio of the right ventricular wall weight to the left ventricular wall plus septum weight
RVSP: Right ventricular systolic pressure
TNF-α: Tumor necrosis factor-alpha
VEGF: Vascular endothelial growth factor
